# Bright Spots in the Darkness of Cancer: A Review of Starfishes-Derived Compounds and Their Anti-Tumor Action

**DOI:** 10.3390/md17110617

**Published:** 2019-10-29

**Authors:** Valentina Lazzara, Vincenzo Arizza, Claudio Luparello, Manuela Mauro, Mirella Vazzana

**Affiliations:** Department of Biological, Chemical and Pharmaceutical Sciences and Technologies (STEBICEF), University of Palermo, 90128 Palermo, Italy; valentina.lazzara@community.unipa.it (V.L.); vincenzo.arizza@unipa.it (V.A.); claudio.luparello@unipa.it (C.L.); manuela.mauro01@unipa.it (M.M.)

**Keywords:** marine invertebrates, sea-star, anti-cancer activity, molecular drugs, natural compounds

## Abstract

The fight against cancer represents a great challenge for researchers and, for this reason, the search for new promising drugs to improve cancer treatments has become inevitable. Oceans, due to their wide diversity of marine species and environmental conditions have proven to be precious sources of potential natural drugs with active properties. As an example, in this context several studies performed on sponges, tunicates, mollusks, and soft corals have brought evidence of the interesting biological activities of the molecules derived from these species. Also, echinoderms constitute an important phylum, whose members produce a huge number of compounds with diverse biological activities. In particular, this review is the first attempt to summarize the knowledge about starfishes and their secondary metabolites that exhibited a significant anticancer effect against different human tumor cell lines. For each species of starfish, the extracted molecules, their effects, and mechanisms of action are described.

## 1. Introduction

Over the years, cancer has remained the second leading cause of death worldwide [1]. Cancer is a complex disease, which arises from an interplay between genetic and environmental factors and characterized by the uncontrolled growth and spread of cells that acquire the ability to overcome cell death, by evading apoptosis. The loss of apoptotic control allows cancer cells to survive longer and to accumulate a huge number of mutations which can improve the invasive capability during tumor progression, promote angiogenesis, deregulate cell proliferation, and interfere with differentiation [2]. Due to its peculiar characteristics, the cancer rate endures despite all the attempts to prevent tumor disorders and the development of new therapies. Thus, the fight against cancer is the main challenge for researchers all over the world, which make every effort to maximize tumor control, prolong survival and improve quality of life for patients [3]. However, the prompt development of resistance to chemotherapeutic drugs, beside the high toxicity usually associated with some cancer chemotherapy drugs and their adverse side-effects, leads to the needs to search for novel anti-tumor compounds, capable of overcome these problems. In this regard, natural products seem to be an attractive source for the development of new drugs, thanks to their capability to strike multiple targets with reduced side effects and their effectiveness against several cancer types [4,5]. Natural compounds derive from various sources such as plants, animals, and microorganisms. Most of them have been approved and play a key role in the treatment of human diseases [6].

Recently, natural compounds of marine origin have given rise to emerging interest in researchers. The oceans constitute the largest habitat of the earth, rich of organisms with high biological and chemical diversity. An interesting branch of pharmaceutical sciences, marine pharmacology, focuses on the substances derived from marine species of plants and animals with active pharmacological properties [7], generally not found in terrestrial natural products. Hence, due to the hostile characteristics of marine environment, such as extreme temperatures, continuous variations in salinity and pressure and the overcoming effects of mutation, bacteria, and viral pathogens, marine organisms developed appropriate mechanisms of adaptation. One of them is the production of biologically active secondary metabolites, which can be considered potent chemical weapons that marine organisms release if necessary [8,9]. Echinoderms are marine invertebrates widely diffused all over the world. There are approximately 7000 extant echinoderm species, included into five well-defined taxonomic classes: Crinoidea (sea lilies and feather stars); Asteroidea (starfishes); Ophiuroidea (basket stars and brittle stars); Holothuroidea (sea cucumbers), and Echinoidea (sea urchins, sand dollars, and sea biscuits) [10]. Echinoderms are an important resource of natural products, which showed to have a significant positive impact on human health. Among them, one of the best examples is surely represented by sea cucumbers, a very promising group of marine invertebrates that during these last decades have gained the attention from researchers worldwide. The active compounds found in these animals are various and include polysaccharides, phospholipids and glycolipids and especially triterpene glycosides or saponins. A lot of studies have revealed their excellent biological effects and nutritive value, their beneficial influence on human health, and pharmacological potential, as recently reviewed by Khotimchenko and Pangestut [11,12]. The present review aims to focus the attention onto another interesting group of echinoderms, the starfishes or sea stars, which represent one of the most promising sources of natural compounds thanks to their variety of species and the value of their products. The class Asteroidea includes approximately 1600 extant species and according to the classification of Blake (1987), seven orders are known, Brisingida, Forcipulatida, Notomyotida, Paxillosida, Spinulosida, Valvatida and Velatida, beside two extinct ones, Calliasterellidae and Trichasteropsida. Starfishes are distributed in all the world’s oceans at all depths, from the intertidal to the abyssal [13]. They are characterized by a star-shaped body plan consisting of a central disc and multiple radiating arms, the number of which varies from 5 to 40. Their bony calcified skin protects them from most predators, and usually they have impressive colors that disguise them or frighten away potential enemies. Moreover, as mentioned before, the presence of particular substances in the sea star’s body represents another important defense strategy. Steroidal glycosides are the predominant metabolites present in starfishes. These compounds have been subdivided into three main groups based on their chemical structures: oligoglycosides (known as asterosaponins), cyclic steroidal glycosides, and glycosides of polyhydroxylated steroids. Polyhydroxysteroidal glycosides from starfish possess peculiar structural characteristics, which is a polyhydroxylated steroidal aglycone (displaying four to nine hydroxyl groups) linked to one or two monosaccharide units [14,15]. Steroidal glycosides from starfishes, especially asterosaponins, showed a wide spectrum of biological activities, including cytotoxic, hemolytic, antibacterial, anti-inflammatory, antitumor and cancer-preventing effects [16,17].

The goal of this review is to present and summarize the information relative to natural compounds derived from starfishes that demonstrated a relevant cytotoxic and anti-tumor effect against different types of human cancer cell lines. Their effects and mechanisms of action are also presented if reported by authors. For each species, the general characteristics, the extracted molecules and their biological potential will be described. An overview of the tested molecules is showed in Table 1.

## 2. *Acanthaster planci* (Valvatida: Acanthasteridae)

**Figure 1:**
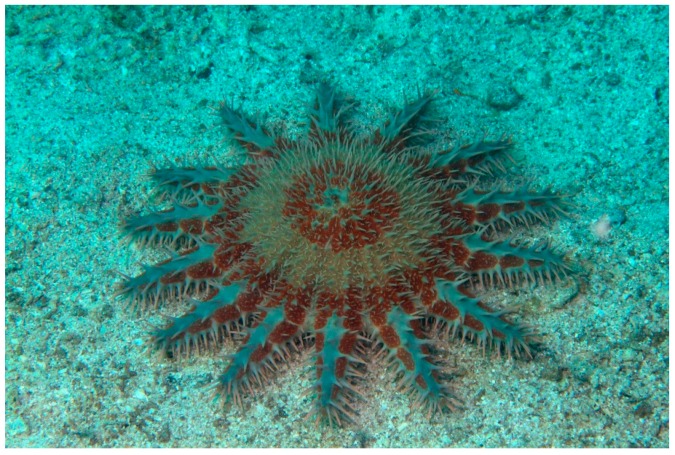
*Acanthaster planci* [19]. © Craig Howe (CC BY-NC-ND).

The starfish *Acanthaster planci* (Linnaeus, 1758) (Figure 1) lives in tropical waters of the Indian and Pacific Oceans. It is one of the largest starfishes, also known as “crown of thorns” because its body possesses numerous large, venomous spines [18]. Its colour varies from red and orange to purple and is thought that these variations could be the result of differences in diet.

The crude extract of *A. planci* was tested against human breast cancer MCF-7 cell line by Mutee et al. [20]. The study demonstrated that the extract has potent cytotoxic as well as apoptotic effects on human breast cancer MCF-7 cell lines compared to tamoxifen. Thus, the results suggested that the extract of *A. planci* starfish, given its strong cytotoxic effect, could be employed as a chemotherapeutic agent in the treatment of breast cancer. Lee et al. [21] in their study extracted a protein toxin from venom of the crown-of-thorns starfish *Acanthaster planci* (Crown-of-thorns *Acanthaster planci* Venom, CAV), the structure of which was identified as plancitoxin protein. The cytotoxic activity against five cell lines was investigated and the results showed that CAV has an anti-proliferative effect on all cell lines, but in particular on human malignant melanoma A375.S2 cells. Investigations about the mechanism of action suggested that CAV reduce cell viability by inducing apoptotic events, which are mediated by mitochondrial membrane and p38 pathways. Moreover, plancitoxin I inhibits the proliferation of A375.S2 cells by inducing oxidative stress, mitochondrial dysfunction and Endoplasmic Reticulum stress-associated apoptosis [22].

Four different fraction of *A. planci* extract (ethanol, non-polar, ethylacetate and butanol fractions) were used to investigate their antioxidant and anticancer properties. Among them, the ethanol fraction showed the highest antioxidant effect, while the butanol fraction induced a significant proliferation inhibition in human malignant melanoma A375.S2 cells. Moreover, further analysis demonstrated that butanol fraction induces both apoptosis and necrosis in A375.S2 cells, evidenced by the upregulation of caspase-3 activity compared to the control and by a significant increase of inter-nucleosomal DNA fragmentation. Thus, *A. planci* can be considered as an important source of biologically-active substances with relevant antioxidant and anticancer effects [23].

New steroid biglycosides, named plancisides A, B, and C were isolated from the ethanolic extract of *A. planci* [18]. Plancisides A exhibited a slight inhibition of cell proliferation of three cancer cell lines (HCT-116, T-47D, and RPMI-7951), but had no relevant effects on colony formation. More recently, the biological activity of a new asterosaponin, called acanthaglycoside G, was investigated along with that of three already known steroidal oligoglycosides (Pentareguloside G, Acanthaglycoside A, Maculatoside) from *A. planci* [24]. An 3-(4,5-dimethylthiazol-2-yl)-5-(3-carboxymethoxyphenyl)-2-(4-sulfophenyl)-2H-tetrazolium) (MTS) assay showed that Acanthaglycoside G and Pentareguloside G exert a negligible cytotoxic effect against human melanoma RPMI-7951, human colorectal carcinoma HT-29, and human breast adenocarcinoma MDA-MB-231 cell lines, while Acanthaglycoside A and Maculatoside slightly inhibited cell viability of HT-29 and MDA-MB-231 cell lines. However, the same compounds exhibited an effective inhibition of the colony formation of cell lines and the ability to prevent the migration of MDA-MB-231 cells, known to have a high metastatic potential.

## 3. Anthenea (Valvatida: Oreasteridae)

*Anthenea chinensis* (Gray, 1840) is an abundant starfish distributed in South China Sea and East China Sea. Ma and collaborators [25] isolated a peculiar polyhydroxysteroidal glycoside, named Anthenoside A, from the starfish *A. chinensis*. It exhibited a potent cytotoxic activity against human leukemia K-562, hepatoma BEL-7402 and glioblastoma U87MG cells, beside the tubulin polymerization-promoting activity. A later study [26] led to the isolation of ten new polyhydroxysteroidal glycosides from the same starfish *A. chinensis*. The extracted compounds, called Anthenosides B–K, were tested against three human tumor cell lines. Most of them showed an inhibitory activity against human leukemia K-562 and human hepatoma BEL-7402 cell lines. Furthermore, the mixture of two compounds exhibited also cytotoxicity against human spongioblastoma U87MG cell line and promoted tubulin polymerization.

In recent years another research group isolated six polyhydroxysteroidal glycosides (anthenosides S1–S6) from the methanolic extract of the starfish *Anthenea sibogae* (Döderlein, 1915). The cytotoxic assay showed a slight inhibition of the proliferation of human breast cancer T-47D cell line by the mixture of two extracted compounds [27].

**Figure 2:**
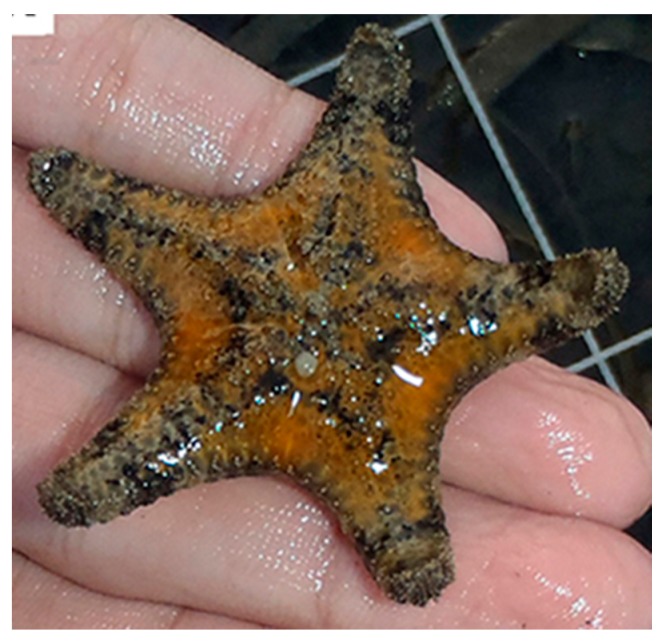
*Anthenea aspera* [28].

In a further study [14] the isolation of new steroidal glycosides from the starfish *Anthenea aspera* (Döderlein, 1915) was carried out (Figure 2), and their in vitro effects on colony formation, cell viability, and apoptosis promotion in three different types of human tumor cell lines, i.e., human melanoma RPMI-7951, breast adenocarcinoma T-47D and colorectal carcinoma HT-29, were evaluated. All the tested compounds showed inhibitory effect on colony formation at nontoxic concentration in the three cell lines. Furthermore, the mixture of two molecules (anthenosides J and K) showed a significant anticancer effect through the induction of apoptosis. Indeed, these compounds cause the downregulation of anti-apoptotic protein expression (Bcl-XL) and the up-regulation of the pro-apoptotic protein expression (Bax and Bak), leading to the activation of the initiator caspase-9 and, in turn, of the effector caspase-3.

Furthermore, two polyhydroxysteroidal glycosides, anthenosides A1 and A2, along with the known anthenoside A, were isolated from *A. aspera*. They contain a 2-acetamido-2-deoxy-4-O-methyl- β-D-glucopyranosyl residue, not previously found in the starfish steroidal glycosides. All the tested compounds exhibited a slight inhibition of cell viability of human cancer T-47D cells, while lacking cytotoxicity against RPMI-7951 cells. Moreover, Anthenosides A1 inhibited colony formation of RPMI-7951 cells moderately if compared to Anthenosides A2 that showed a reduction of the number of colonies of T-47D cells down to 40% [29].

## 4. Archaster typicus (Valvatida: Archasteridae)

**Figure 3:**
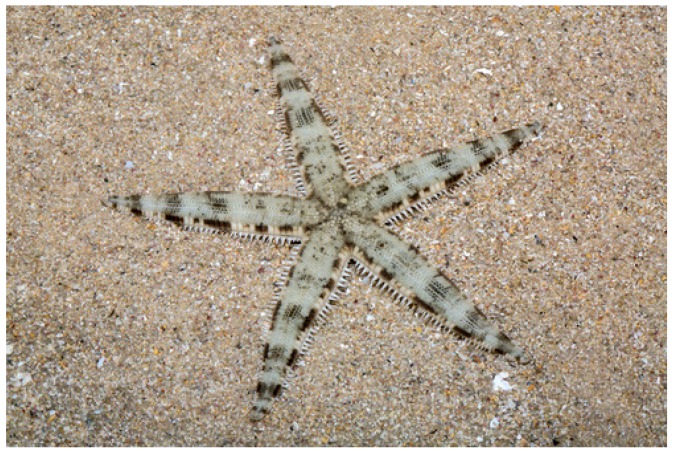
*Archaster typicus* [31]. © budak. Some rights reserved (CC-BY-NC).

*Archaster typicus* (Müller & Troschel, 1840) is distributed in the shallow waters of the Western Indian Ocean and the Indo-Pacific. Also known as a sand star, it has five long, slightly tapering arms with pointed tips [30]. It has usually a grey or brownish colour, variously marked with darker and lighter patches (Figure 3).

Kicha et al. [32] isolated new asterosaponins, named Archasterosides A and B, and the known regularoside A from the starfish *A. typicus* and their anticancer properties were evaluated. The 3-(4,5-dimethylthiazol-2-yl)-5-(3-carboxymethoxyphenyl)-2-(4-sulfophenyl)-2H-tetrazolium) MTS assay revealed that all three compounds display a moderate anticancer activity and cytotoxicity against human cancer HeLa cells and mouse epidermal JB6 P + Cl41 cells, even if with different IC50. Moreover, since Archasterosides B exhibited the highest restraining potential compared to the other two tested compounds, further analyses were performed to investigate its effect on the basal AP-1-, p53, and NF-κB-dependent transcriptional activities, using JB6 Cl41 cells. The result showed for the first time that Archasterosides B induced basal p53- and AP-1-, but not NF-κB-dependent transcriptional activities compared to untreated controls.

## 5. Asterias amurensis (Forcipulatida: Asteriidae)

**Figure 4:**
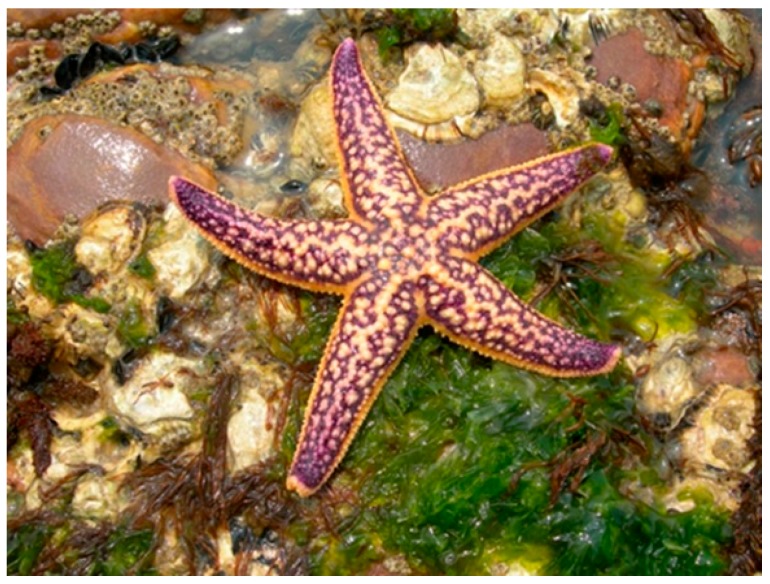
*Asterias amurensis* [34].

*Asterias amurensis* (Lutken, 1871) is commonly known as Northern Pacific sea star or Japanese sea star (Figure 4). It is widely distributed in estuarine, intertidal, and coastal zones of the Pacific Ocean near Japan, Russia, Northern China, and Korea, but this species habits also the southern Australian coast (especially Tasmania), Alaska and the Aleutian Islands, Europe, and the state of Maine. *A. amurensis* has five arms, which joins in the center of the animal forming a central disc. Below each arm run irregularly arranged spines. They also line the ventral groove of each arm, where the tube feet are found. This starfish is characterized by a variety of colours, ranging from orange to yellow, or sometimes purple on their dorsal side [33].

Lei et al. [35] examined the anti-tumor activity of cerebrosides derived from the sea cucumber *Acaudina molpadioides* (AMC) and the starfish *Asterias amurensis* (AAC), both in vitro and in vivo. Cerebrosides are one of the simplest classes of glycosphingolipids that are widely present in the plasma membranes of fungi, plants and marine organism. They are characterized by monosaccharides, an amide-linked fatty acid and a sphingoid base commonly named long-chain base [36] and in several studies they showed to play a series of biological roles [37,38,39]. In particular, these molecules were tested in vitro against murine sarcoma cells (S180) and the results obtained demonstrated a dose-dependent inhibitory effect of AAC-derived cerebrosides on cell proliferation, which appears stronger and more efficient than that of AMC-derived ones. Further investigations revealed that this restraining effect occurs through the mitochondria-mediated apoptosis pathway, via the downregulation of Bcl-2 and Bcl-XL expression and the up-regulation of Bax, Cytochrome c, caspase-9 and caspase-3 expression. In addition, a significant reduction of tumor growth was obtained in vivo by treating S180 tumor-bearing mice, although in this case AAC-derived cerebrosides were less potent than AMC-derived ones.

## 6. Asterina pectinifera (Valvatida: Asterinidae)

**Figure 5:**
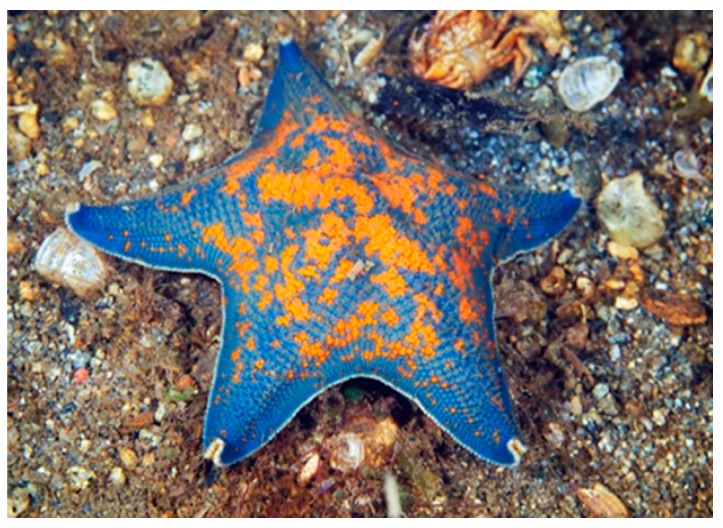
*Asterina pectinifera* [40]. © Alexander Semenov.

*Asterina pectinifera* (Muller & Troschel, 1842), also known as blue bat starfish, is native to the Yellow Sea, Sea of Japan and Est Sea but it habits also the coasts of Russia. It has five large and short arms and presents a vivid color (blue) with some orange spots (Figure 5). Usually *A. pectinifera* prefers the shallow zones of the sea and feeds on small invertebrates, algae, and detritus.

Lou et al. [41] in their study tested the biological activity of a series of peptides derived from the motifs of cyclin B of *A. pectinifera*. Interestingly, the complex CDK1/cyclin B of this starfish, described by McGrath [42], showed a significant similarity with the human CDK2/cyclin A complex. Therefore, the authors investigated the biological potential of three selected motifs of cyclin B (α5 helix, α3 helix and N-terminal, named a5, a3 and N respectively) on two human tumor cell lines, i.e., HCT-116 (human colon adenocarcinoma cells) and EC-9706 (esophageal carcinoma cells). To facilitate the cellular uptake of these peptides, they were conjugated with the protein transduction domain of HIV-1 transactivator of transcription (Tat). The results showed a significant cytotoxic activity of Tat-a5 in both cell lines, compared to the other peptides examined. Further investigations suggested that Tat-a5 compete with Cyclin B1 and bind to CDK1 preventing its excessive activation. As a consequence, cancer cells get arrested at G2/M phase and go toward apoptosis. These considerations suggest that the peptide Tat-a5 could be a novel relevant compound with anticancer and tumor angiogenesis-inhibiting activity.

Peng and his collaborators [43] isolated a new polyhydroxysterol ester and seven known steroid derivatives from *A. pectinifera*. They investigated the cytotoxic potential of these molecules and the results showed that only two compounds (25S)-5α-cholestane-3β,4β,6α,7α,8,15β,16β,26-octol and Cholest-7-en-3-sodium sulfate had a marginal antitumor activity on human liver carcinoma HepG2 cells in vitro.

Malyarenko et al. [44] recently investigated the anticancer and radiosensitizing activities of polar steroids from *A. pectinifera* in human colorectal carcinoma cells DLD-1, HCT 116, and HT-29. Among the tested compounds, (25S)-5α-cholestane-3β,4β,6α,7α,8,15β,16β,26-octaol and Asterosaponin P1 showed the greatest results in terms of cytotoxic activity. Moreover, Asterosaponin P1 strengthened the efficacy of radiation, leading to the reduction of the number and size of the colonies of colorectal cancer cells. Further experiments demonstrated that the radiosensitizing activity of this compound is given by apoptosis induction via the regulation of anti- and pro-apoptotic protein expression followed by the activation of the initiator and effector caspases (caspase-9 and caspase-3, respectively) and DNA degradation.

Another interesting study of Nam and Shon [45] investigated the effect of polysaccharides extracted from *A. pectinifera*. In a previous study [46] it was demonstrated that polysaccharides extracted from *A. pectinifera* inhibited the cytochrome P450 1A1- mediated ethoxyresorufin O-deethylase activity and reduced significantly the ornithine decarboxylase activity in polysaccharide-treated MCF-7 breast cancer cells. Then, the activity of phase II detoxification enzymes, such as quinone reductase (QR), glutathione S-transferase (GST) and ornithine decarboxylase (ODC) in HT-29 human colon adenocarcinoma cells was investigated [45]. These enzymes are involved in the prevention of carcinogen-induced colon cancer. The results showed that the polysaccharides increased the activity of both QR and GST enzymes. This suggests the presence of some constituents capable to prevent the initiation of colon carcinogenesis through this mechanism. Moreover, the treatment of the HT-29 cells with the polysaccharides extracted from *A. pectinifera* showed a reduced activity of ODC, whose induction stimulate tumor promotion. Further investigations showed the inhibition of TPA-induced COX-2 activity, suggesting a useful application of these polysaccharides in the inhibition of colon carcinogenesis.

The chemopreventive effect of this type of extract was also demonstrated by Lee et al. [47]. They investigated the biological activity of *A. pectinifera* polysaccharides on the progression and metastasis of human breast cancer. The results showed for the first time the ability of starfish polysaccharides to suppress carcinogenesis and dissemination of human breast cancer through the decrease of COX-2 and aromatase expression and the inhibition of cell motility. Thus, these promising results suggest that starfish polysaccharides might be valuable chemopreventive agents.

Moreover, Kim and collaborators in another recent study [48] demonstrated that the *A. pectinifera* fermented with *C. militaris* mushroom mycelia (FACM) displayed a strong anti-cancer activity, compared to unfermented *A. pectinifera*. Indeed, FACM was able to decrease B16F10 murine melanoma cell proliferation significantly, increasing in parallel the activation of apoptosis by increasing levels of the pro-apoptotic protein Bax and reducing the levels of the anti-apoptotic protein Bcl-2. This means that FACM could be a promising anti-cancer agent, but further studies are needed to identify the bioactive substances responsible of these biological effects.

## 7. Asteropsis carinifera (Valvatida: Asteropseidae)

**Figure 6:**
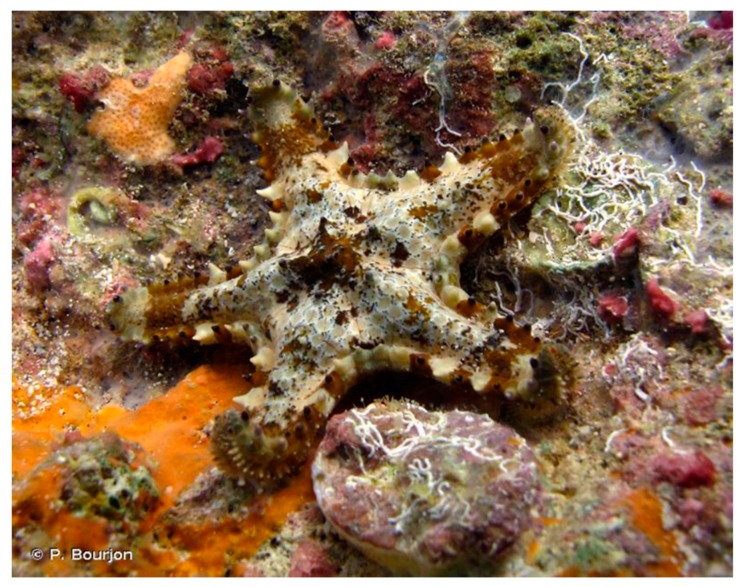
Asteropsis carinifera [49]. © P. Bourjon.

The starfish *Asteropsis carinifera* (Lamarck, 1816) is distributed in the Indo-Pacific area. It has five arms characterized by the presence of large conical and dull spines aligned in the middle and on the periphery of each arm. The body is relatively flat but slightly convex towards the middle of the central disk and the colour is usually gray with stained brown spots (Figure 6).

Six steroidal biglycosides, named Cariniferosides A–F, along with six previously known glycosides, were isolated from the alcoholic extract of the starfish *A. carinifera* in a study of Malyarenko et al. [50]. The isolated compounds were evaluated for their biological activities on human breast cancer cells T-47D, human malignant melanoma cells RPMI-7951, and human colon cancer cells HCT-116. The results did not show cytotoxicity in any of the cancer cell lines tested. However, three compounds, compared to the others, significantly inhibited colony formation of RPMI-7951 and T-47D cell. Subsequently, a new asterosaponin, Asteropsiside A, was isolated from the starfish *A. carinifera* and the biological effect of two previously known asterosaponins, Regularoside A and Thornasteroside A were evaluated [51]. The cytotoxicity assays demonstrated that both glycosides exhibit toxic properties against HCT-116, RPMI-7951, and T-47D cancer cell lines. Moreover, a clonogenic assay showed also the ability to inhibit the rate of colony formation of tumor cells in vitro.

## 8. Astropecten (Paxillosida: Astropectinidae)

**Figure 7:**
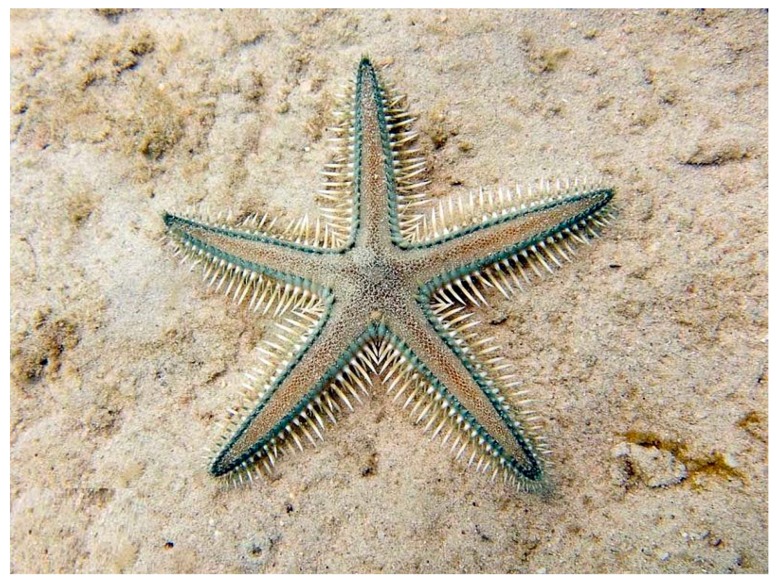
Astropecten polyacanthus [52]. © Chaloklum Diving CC-BY-3.0 (https://commons.wikimedia.org).

The genus *Astropecten* includes a huge amount of species, very similar to each other, that inhabit sandy or muddy seabeds. One of them, *Astropecten polyacanthus* (Müller & Troschel, 1842) is commonly known as the Sand Sifting Starfish (Figure 7). It is an innocuous invertebrate, distributed in the Indo-Pacific Ocean. It possesses teeth or spine like structures running along the outer edges of its five arms, which help the sea star to conduct its benthic life, by moving, burrowing, and feeding.

Thao et al. [53] in their study isolated four steroids, called Astropectenols A-D, along with three known compounds (5-7) from *A. polyacanthus*. They evaluated the anti-cancer activity of the methanol extract and of the isolated compounds on three human cancer cell lines, HL-60 (leukemia cells), PC-3 (prostate cancer cells) and SNU-C5 (colorectal cancer cells). The extract of the starfish showed a potent cytotoxic activity against leukemia cells and prostate cancer cells, while only the compound 7 exhibited a relevant cytotoxicity compared to the others. Further investigations suggested that the inhibitory activity is due to the induction of apoptosis, mediated by the regulation of apoptosis-related protein expression (i. e. down-regulation of Bcl-2, up-regulation of Bax, cleavage of caspases-9, caspases-3 and PARP) and via the downregulation of ERK1/2 pathway and of the oncoprotein C-myc.

A recent study of Vien [54] investigated the cytotoxicity of five polar steroid derivatives, including one new glycosylated polyhydroxysteroid named polyacanthoside, isolated from the water-soluble materials from the MeOH extract of *A. polyacanthus*. Only (20R,24S)-3β,6α,8,15β,24-pentahydroxy-5α-cholestane exhibited significant cytotoxic effect against five human cancer cell lines as HepG2 (hepatoma cancer), KB (epidermoid carcinoma), LNCaP (prostate cancer), MCF7 (breast cancer), and SK-Mel2 (melanoma). The compounds Marthasteroside B and Psilasteroside had moderate effects on all five cell lines, whereas Polyacanthoside A and Triseramide appeared to display an even lesser activity.

**Figure 8:**
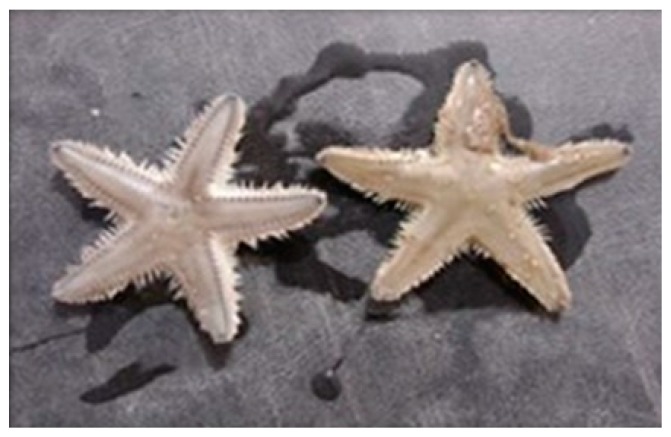
*Astropecten monacanthus* [55].

The starfish *Astropecten monacanthus* (Sladen, 1883) (Figure 8) habits the benthic tropical zone of the Indo-Pacific Ocean.

Thao et al. [56] in their study isolated six asterosaponins from *A. monacanthus*, whose structure and anti-inflammatory activity were elucidated in a previous work [57]. Both the whole extract and the isolated compounds were tested against three human cancer cell lines (HL-60, PC-3, SNU-C5) to check their cytotoxic potential. The results showed a potent cytotoxic activity of Astrosterioside D on all cell lines. Furthermore, the methanol extract exhibited a more potent cytotoxic activity compared to the isolated saponins. This suggested that the inhibitory activity could be due to the synergic effect of more than one compound. Further investigations clarified that the induction of apoptosis through the downregulation of PI3K/AKT and ERK 1/2 MAPK pathways is responsible of the cytotoxic activity.

## 9. Certonardoa semiregularis (Valvatida: Ophidiasteridae)

*Certonardoa semiregularis* (Muller & Troschel, 1842) is widely diffused in the Eastern China Sea and Japan. It has five slender arms broad at base, cylindrical in cross-section, and tapering to rounded tips. The dorsal surface is usually red and characterized by plates that are organized in regular longitudinal and transverse rows, while the ventral surface is pale [58].

Wang and his group [59], isolated a series of biomolecules from *C. semiregularis* and tested their cytotoxic activity against a panel of human solid tumor cell lines (A549, SK-OV-3, SK-MEL-2, XF498, HCT15). The polyhydroxysterols, named Certonardosterols, showed moderate to significant cytotoxicity against these cell lines. Four new saponins, designated as Certonardosides K – N, were evaluated for their cytotoxic activities along with the previous extracted compounds, Certonardosides A–J [60,61]. Among them, certonardoside C was the most active against the SK-MEL-2 skin cancer cell line. Further studies [62,63] led to the isolation of a significant number of new polyhydroxysterols and ten new saponins, which showed again a considerable cytotoxic activity against a series of human tumor cell lines. In particular, a rare example of 15-keto steroid exerted the highest cytotoxic effect. Moreover, two new sulfated saponins, Certonardosides P2 and I3, were isolated from the brine shrimp active fraction of the methanolic extract of *C. semiregularis*. These compounds were tested for their cytotoxic potential against five human tumor cell lines (A549, SK-OV-3, SK-MEL-2, XF498, and HCT15), but only Certonardosides P2 displayed a relevant cytotoxicity against the SK-MEL-2 skin cancer cell [58].

## 10. Choriaster granulatus (Valvatida: Oreasteridae)

**Figure 9:**
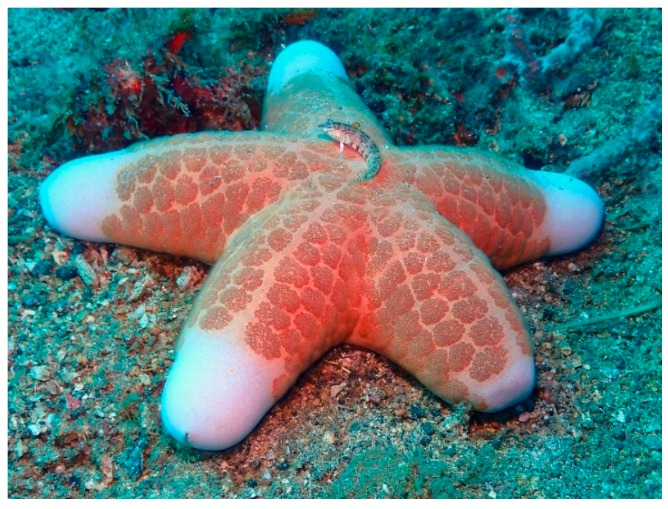
Choriaster granulatus [64]. © Mark & Marta Bockstael-Rubio (https://www.jungledragon.com).

*Choriaster granulatus* (Lütken, 1869) habits the Indo-West Pacific waters (Figure 9). It is found in sandy zones, among corals and sponges and feeds on coral polyps and other small invertebrates. This starfish has a large convex body with five short arms. It has a typical pale pink color characterized by brown papillae in the center.

Recently, Ivanchina et al. [65] by continuing their research about *C. granulatus*, isolated sixteen polar steroids, along with two new polyhydroxy glycosides, granulatosides D and E, from the ethanolic extract of the starfish. The investigations on the cytotoxic potential of these compounds demonstrated that only four of the tested compounds (Granulatosides D, Echinasterosides F, desulfated Echinasteroside B and Laeviuscoloside D) exhibited a significant cytotoxic activity against murine splenocytes.

## 11. *Craspidaster hesperus* (Paxillosida: Astropectinidae)

**Figure 10:**
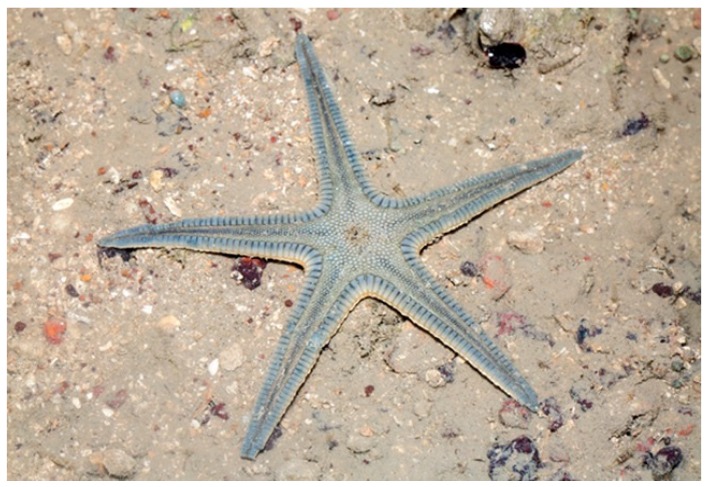
*Craspidaster Hesperus* [67]. © budak, Some Right Reserved (CC BY-NC).

*Craspidaster hesperus* (Muller & Troschel, 1840) (Figure 10) is distributed in the Indo-West Pacific Ocean. It is a flat sea star with five elegant tapered arms. The dorsal surface of its body presents special flat-topped, pillar-like structures, called paxillae. Large marginal plates characterize the body’s edges and the tube feet are pointed [66].

Kang et al. [15] isolated three polyhydroxysteroidal glycosides (hesperuside 1–3) from the starfish *Craspidaster hesperus*. These compounds exhibited cytotoxic activity against three types of human cancer cells (human leukemia MOLT-4, human hepatoma BEL-7402 and human lung cancer A-549). Interestingly, it was supposed a possible correlation between the cytotoxicity and the structural features of the steroidal glycosides.

## 12. Ctenodiscus crispatus (Paxillosida: Ctenodiscidae)

**Figure 11:**
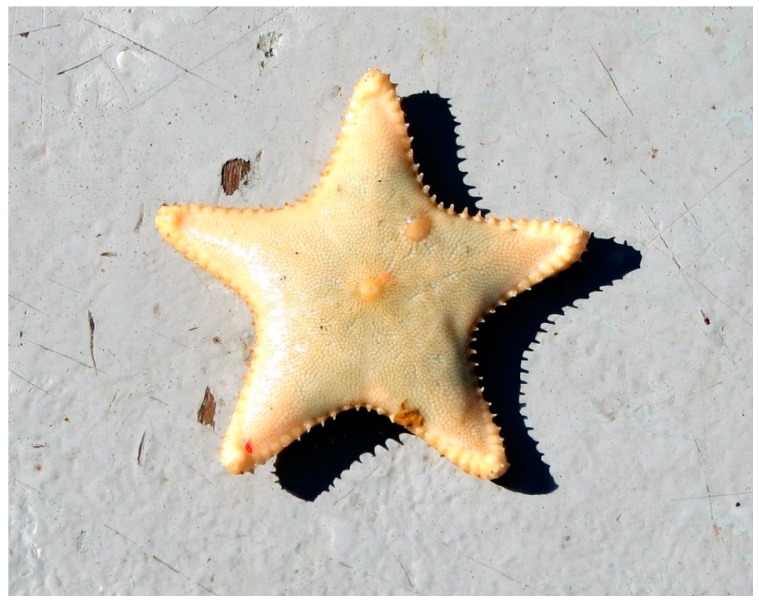
*Ctenodiscus crispatus* [69]. © Rick (https://www.flickr.com).

The starfish *Ctenodiscus crispatus* (Bruzelius, 1805) (Figure 11) is broadly distributed worldwide, especially on mud bottoms of northern coasts [68]. It has rounded short and large arms, the dorsal side is large, usually yellow-orange, while the ventral side is whitish. This starfish is characterized by the presence of vertical row of spikes and cilia along the edge of arms that form the so-called cribriform organ. This helps to increase the flow of oxygen rich water across the body when it is covered in the mud where the starfish live.

Quang and collaborators [70] isolated and identified five steroids from *C. crispatus* and investigated the biological potential of these compounds on two cell lines from human hepatocellular carcinoma (HepG2) and human glioblastoma (U87MG). Among the five, only one of these compounds, the (25S)-5α-cholestane-3β,5,6β,15α,16β,26-hexaol, showed relevant biological activity. It exhibited a clear reduction in cell viability of both cell lines in a dose-dependent manner. The following investigations performed to elucidate the mechanism responsible of cell death suggested that the cytotoxic effect of this compound could be derived from the induction of apoptosis, through an up-regulation of the ratio of Bax:Bcl-2, followed by the release of cytochrome c from the mitochondria and the associated activation of caspases and PARP.

## 13. Culcita novaeguineae (Valvatida: Oreasteridae)

**Figure 12:**
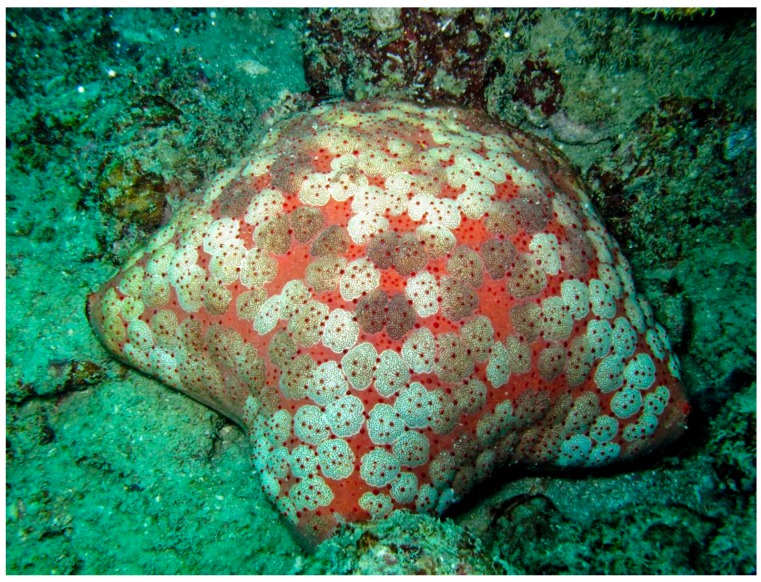
*Culcita novaeguineae* [71]. © Western Australian Museum Collections (CC BY 4.0).

*Culcita novaeguineae* (Müller & Troschel, 1842), commonly known as pin cushion star, is pentagonal in shape, with short arms and an inflated appearance (Figure 12). The ventral side presents a central mouth and rows of tube feet. *C. novaeguineae* is characterized by variable colours, and it usually habits the coral reefs of the warm water in the Indo-Pacific Ocean.

Two asterosaponins, named novaeguinosides I and II, together with a known saponin, regularoside B, were isolated from the starfish *Culcita novaeguineae* [72]. The cytotoxic activity of these compounds was tested, and the results showed a marginal effect against two human tumor cell lines (human leukemia K-562 cells and human hepatoma BEL-7402 cells).

Zhou and collaborators [73] in their study investigated the biological activity of novaeguinoside II on human U87MG glioblastoma cell line. This compound showed a relevant suppression of U87MG cell proliferation due to the activation of mitochondrial apoptotic pathway, characterized by the up-regulation of cytochrome-c and caspase-3 expression. Another study [74] led to the isolation of novel asterosaponins. Among them, Asterosaponin 1 and 3 showed a significant cytotoxic activity against two human tumor cell lines, i.e., leukemia K-562 and hepatoma BEL-7402.

Subsequently, Asterosaponin 1 was proven capable to inhibit the growth of human glioblastoma cells through the mechanism of apoptosis, in a dose- and time-dependent manner [75]. These findings suggest the employment of Asterosaponin 1 as a promising novel anticancer drug. Some years later, four novel asterosaponins, named novaeguinosides A-D, were isolated from *C. novaeguineae* and demonstrated the capability of promoting the polymerization of tubulin in vitro [76]. Moreover, the evaluation of the in vitro cytotoxic activity of these new asterosaponins showed great results against K-562 and BEL-7402 cells. Two asterosaponins, including a new compound named Novaeguinoside E, and six glycosylated polyhydroxysteroids were isolated from a methanol extract of the starfish *C. novaeguineae* [77]. The investigation about their biological activity against five human cancer cell lines demonstrated that halytilosided B and D have a moderate cytotoxic activity against KB (epidermoid carcinoma), LNCaP (prostate cancer), MCF7 (breast cancer), and SK-Mel2 (melanoma); halytiloside A showed a moderate cytotoxicity against LNCaP and MCF7 cells, but a weaker effect on KB and SK-Mel2 cells. Culcitoside 5 exhibited a weak cytotoxic activity against KB, LNCaP, MCF7 and SK-Mel2 cell lines. Recently, the cytotoxic activity of new polyhydroxy steroidal glycosides, named culcinosides A-D, against human glioblastoma cell lines was evaluated [78]. Among them, culcunoside A showed a significant activity, while the others demonstrated a moderate cytotoxicity.

## 14. Echinaster luzonicus (Spinulosida: Echinasteridae)

**Figure 13:**
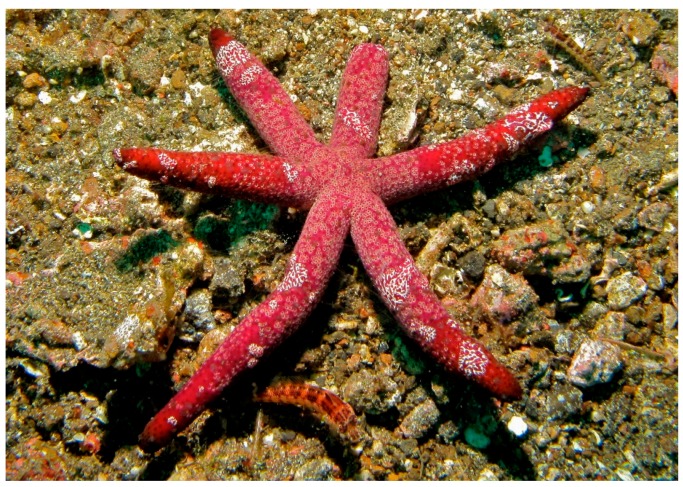
*Echinaster luzonicus* [79]. © Bernard Dupont (CC-BY-SA-2.0).

The starfish *Echinaster luzonicus* (Gray, 1840) populates the tropical and sub-tropical western Indo-Pacific area. It has six thin arms and its colour range from red and brown, according to the surrounding environment in which they live (Figure 13).

In recent years, Malyarenko [80] tested two cyclic steroidal glycosides, Luzonicoside A (LuzA) and D (LuzD) extracted from the starfish *E. luzonicus*, against human RPMI-7951 and SK-MEL-28 melanoma cells. The in vitro inhibitory activity of these molecules and their mechanism of action were investigated. The results showed a significant anti-cancer activity induced by cell cycle regulation and apoptosis; in particular, the up-regulation of p21 and the downregulation of the expressional levels of Cyclin D1 lead to cell cycle arrest, while the downregulation of Bcl-2 protein and the activation of the effector caspase-3 determine the apoptotic event. Moreover, LuzA revealed a major capability of inhibiting the proliferation, the migration and the formation of colonies of cancer cells compared to LuzD. However, the overall results suggest a promising development of both LuzA and D as anti-cancer drugs.

## 15. Henricia leviuscula (Spinulosida: Echinasteridae)

**Figure 14:**
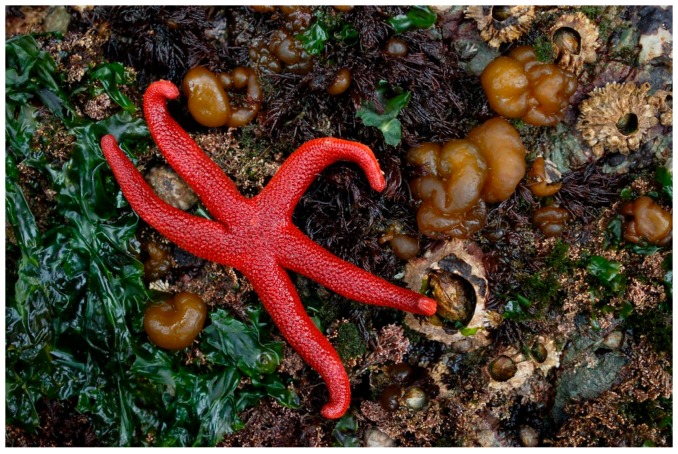
*Henricia leviuscula* [81]. © Christopher Lindsey.

*Henricia leviuscula* (Stimpson, 1857), commonly known as the Pacific blood star, is a starfish that habits the Pacific coast of North America. It has a brilliant red-orange color and five, long, tapering arms, characterized by the absence of pedicellariae and spines (Figure 14).

An interesting study of Fedorov [82] reports the evaluation of the proapoptotic and anticarcinogenic properties of Leviusculoside G, a glycosylated steroid isolated from *H. leviuscula*. The results revealed that the treatment of human leukemia HL-60, THP-1, and mouse skin JB6 Cl41 cells with nontoxic doses of Leviusculoside G induced apoptosis and decreased cell transformation. Moreover, preliminary studies aimed to clarify the mechanism of action of this compound demonstrated that Leviusculoside G could exert its effect through the promotion of p53-dependent apoptosis and the inhibition of AP-1, NF-κB, and ERK activities.

## 16. Hippasteria phrygiana (Valvatida: Goniasteridae)

**Figure 15:**
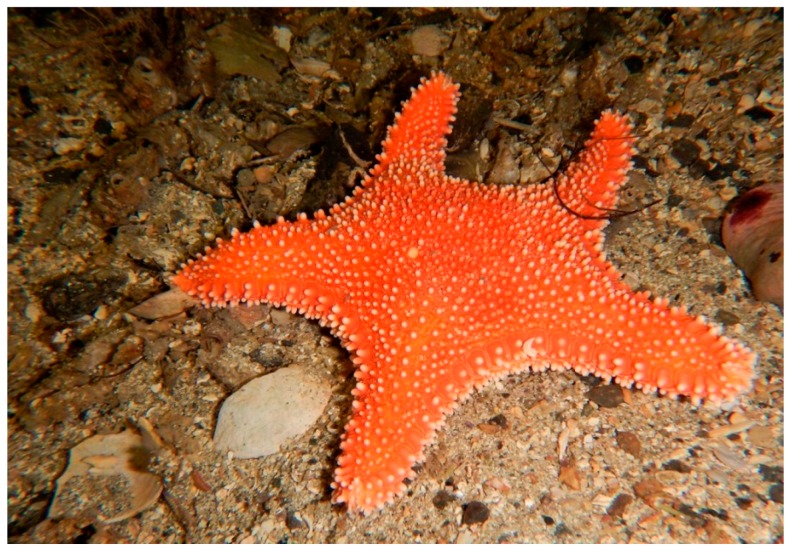
*Hippasteria phrygiana* [83]. © Viktor V. Grøtan, some right reserved (CC BY-NC).

*Hippasteria* represents a group of cold-water starfishes, typically coral/cnidarian predators. They are five-armed sea stars, characterized by a large and flattened central disc. *Hippasteria phrygiana* (Parelius, 1768 from World Register of Marine Species-WoRMS), also named *H. kurilensis*, is widely distributed in the oceans (Figure 15).

Kicha et al. [84] isolated four new asterosaponins, Hippasteriosides A–D, from the alcoholic extract of the Far Eastern starfish *H. kurilensis*. The structures of the compounds were characterized and their cytotoxic activity was evaluated against human colon tumor HT-29 cells. All the compounds did not show a significant effect on cell growth within a 15–120 mg·mL^−1^ concentration range. However, Hippasterioside D at non-cytotoxic concentration (60 mg·mL^−1^) was able to reduce both colony number and size if compared with non-treated cells, while Hippasteriosides A and B exhibited a less pronounced potential than hippasterioside D in inhibiting cell transformation. Hippasterioside C failed to demonstrate any significant effect on HT-29 cells. Thus, Hippasterioside D seems promising for further investigation as an anticancer agent.

Another study [85] led to the isolation of the new cyclopropane-containing steroid Phrygiasterol from the ethanol extract of *H. phrygiana*. It was tested against Ehrlich carcinoma cells, together with another component of the extract called Borealoside C to investigate their biological activity. The results showed a moderate cytotoxic activity of Phrygiasterol against Ehrlich carcinoma cells, while Borealoside induced apoptosis of the same cell line.

## 17. Leptasterias ochotensis (Forcipulatida: Asteriidae)

*Leptasterias ochotensis* (Brandt, 1851) belongs to the genus Leptasterias, whose members are characterised by having six arms.

Malyarenko et al. [86] investigated the biological activity of the asterosaponins leptasteriosides A–F, one new and one previously known asterogenin, extracted from the starfish Leptasterias ochotensis. The compounds exhibited different effects. Only the three asterosaponins Leptasterioside B, Leptasterioside C, and particularly Leptasterioside A showed a significant cytotoxic activity against T-47D cell line. Moreover, Leptasterioside A exhibited also the highest inhibitory effect on colony formation by T-47D breast cancer cell line at low concentration compared to the others. Interestingly, the effects of the compounds on colony formation was also dependent on the type of cell line. Moreover, Leptasterioside A at low concentration was shown to be more effective in inhibition of colony formation of T-47D and RPMI-7951 cells than cisplatin, used as positive control by the authors. Therefore, the overall results indicated that Leptasterioside A could be a promising cancer-preventive and anticancer agent, but further investigations are needed to confirm this hypothesis.

Another study [87] reported the isolation of three new sulfated steroid monoglycosides, called leptaochotensosides A–C, and a new sulfated polyhydroxylated steroid from *L. ochotensis*. The cytotoxicity of the compounds against melanoma RPMI-7951 and breast cancer T-47D cell lines was investigated, but in both cases the molecules were not found able to decrease cell viability. Then, the effect of the compounds on colony formation of human melanoma RPMI-7951 and human breast cancer T-47D cells was also checked. Interestingly, only Leptaochotensoside A reduced the colony formation of T-47D cells in a significant way compared with non-treated controls. In order to better understand the mechanism of action of the molecule, the mouse epidermal JB6 Cl41 cells were used to test the effect of Leptaochotensoside A on EGF-induced colony formation. The results showed a relevant reduction of colony and this cancer preventive action of leptaochotensoside A appeared to be associated with the regulation of mitogen-activated protein kinase (MAPK) signalling pathway (i. e. ERK1/2 and MSK-1 kinases).

## 18. Lethasterias fusca (Forcipulatida: Asteriidae)

Two new asterosaponins, lethasteriosides A and B, along with previously known glycosides, thornasteroside A, anasteroside A and luidiaquinoside were extracted from *Lethasterias fusca* (Djakonov, 1931) by Ivanchina et al. [88]. The cytotoxicity against human breast T-47D, colorectal carcinoma HCT-116, and melanoma RPMI-7951 cancer cell lines was evaluated. The tested compounds did not show significant cytotoxic effect against these cancer cell lines. Anyway, lethasteriosides A, exhibited an interesting inhibition of the colony formation in all the cell lines.

## 19. Narcissia canariensis (Valvatida: Ophidiasteridae)

**Figure 16:**
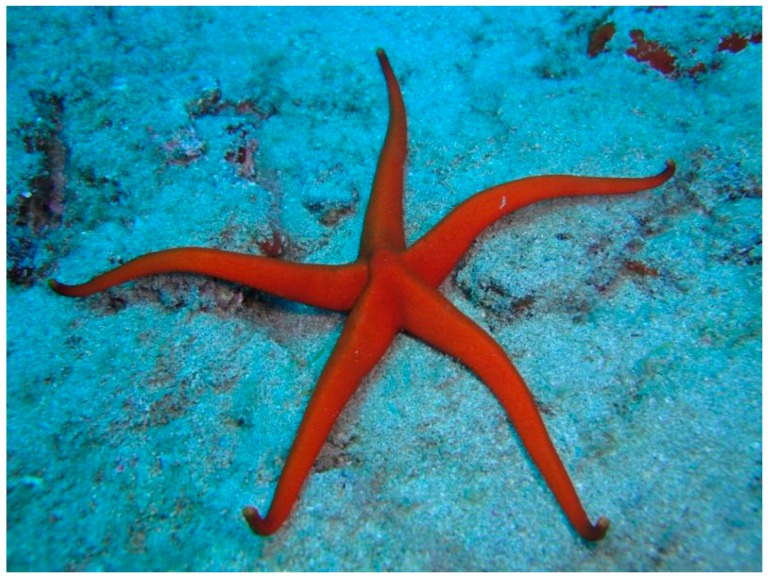
*Narcissia canariensis* [89].

*Narcissia canariensis* (d’Orbigny, 1839) is a five-armed starfish characterized by an even orange colour with a yellow tip at the end of each arm (Figure 16). This species is widely distributed in the Canaries, but it also inhabits the areas of Cape Verde, the Gulf of Mexico, Madeira, the Azores and the Congo.

The glycolipid fraction isolated from the starfish *N. canariensis*, containing particular glycosphingolipids named ophidiacerebrosides, was evaluated for its cytotoxic activity against various human cancer cell lines from multiple myeloma, colorectal adenocarcinoma and glioblastoma multiforme by Farokhi [90]. The results demonstrated that the fraction (F13-3) displayed an interesting cytotoxic activity over 24 h.

## 20. Pentaceraster gracilis (Valvatida: Oreasteridae)

The starfish *Pentaceraster gracilis* (Lütken, 1871) habits the bays of North Australia and the waters of Indian Ocean and Red Sea. It has a peculiar short triangular form from which the long arms depart. The study of Vien et al. [91] reports the isolation of two steroid glycosides namely Pentacerosides A and B, and four know compounds from the methanol extract of the starfish *P. gracilis*. The cytotoxic activity of the isolated compounds against five human cancer cell lines, HepG2 (hepatoma cancer), KB (epidermoid carcinoma), LNCaP (prostate cancer), MCF7 (breast cancer), and SK-Mel2 (melanoma) was investigated. Only maculatoside displayed a significant cytotoxic effect against Hep-G2 and SK-Mel2 cell lines and a moderate effect on KB, LNCaP and MCF7 cell lines, compared to the positive control, ellipticine.

## 21. Focusing on Cytotoxic Potential and the Mechanism of Action

Starfish-derived steroidal glycosides represent an interesting and promising resource for the treatment of cancer. Indeed, the multitude of studies performed during the recent years highlighted the important biological potential of these secondary metabolites extracted from a variety of starfish species. Among them, as already mentioned, polyhydroxysteroidal glycosides and asterosaponins showed the more prominent cytotoxic effect and anti-tumor activity against a great deal of human cancer cell lines. A summary of the most efficient compounds and their biological effects is reported in Table 2 and Table 3.

Once the effect of these compounds was evaluated, the researchers’ attention has focused on the mechanisms of action through which steroidal glycosides exert their biological activities. The investigations revealed that the promotion of apoptosis is the main mechanism behind cytotoxicity. Apoptosis, also known as programmed cell death, is an evolutionary-conserved process involved in organism development and tissue homeostasis. There are two types of apoptosis program, i.e., intrinsic (or mitochondrial) and extrinsic (or death receptor-mediated). Both pathways entail the activation of initiator caspases (9 and 8, respectively), which in turn start a cascade of proteolytic cleavages involving effector caspases responsible for the execution phase of apoptosis.

Effector caspases also act on the integrity of the cytoskeleton, and on components of the cell cycle and signal transduction pathways thereby contributing to the onset of the morphological hallmarks of apoptosis (chromatin condensation, nuclear fragmentation, rounding up of the cell and reduction in cell volume). The apoptotic trigger that activate the mechanism is regulated by the balance between pro-apoptotic and anti-apoptotic members of the Bcl-2 family proteins. Bcl-2, Bcl-xL, Mcl-1, Bcl-w, A1/Bfl-1, and Bcl-B/Bcl2L10 belong to the anti-apoptotic category, while Bax, Bak, and Bok/Mtd are examples of pro-apoptotic proteins. In pathological conditions, such as cancer, cells acquire the ability to escape apoptosis through several mechanisms, leading to their uncontrolled proliferative behaviour. However, the most common molecular basis of the onset of apoptosis is certainly the alteration of the ratio between pro-apoptotic and anti-apoptotic proteins [92,93,94]. Hence, the research and identification of new natural compounds capable to induce the apoptosis in cancer cells is a hopeful approach to reduce or arrest tumor growth. In this context, several studies have emphasized the potential of the examined polyhydroxysteroidal glycosides and asterosaponins to regulate the expression of apoptotic protein. Indeed, these compounds trigger a down-regulation of the expression of anti-apoptotic proteins (e.g., Bcl-2) and an up-regulation of that of pro-apoptotic proteins (e.g., Bax) in cancer cells, as shown by the biological assays. Furthermore, some evidence demonstrated also that steroidal glycosides from starfishes could regulate two pathways, i.e., those involving mitogen-activated protein kinases (MAPKs) and PI3K/AKT, which control several intracellular processes, including apoptosis. Three subfamilies of MAPKs have been identified: extracellular signal-regulated kinase (ERK; ERK1 and ERK2), c-Jun N-terminal kinase (JNK; JNK1, JNK2, and JNK3), and p38-MAP kinase (α, β, δ, and γ), while the PI3K/Akt signaling pathway is one of the best-characterized with regard to the transmission of anti-apoptotic signals in cell survival.

These compounds have very peculiar properties, especially considering that, in bibliography, animals such as starfish are unknown to develop tumors, so it may be surprising the presence of substances capable of exerting this effect. Actually, the secondary metabolites of marine organisms represent “weapons”, defense strategies against extraneous and harmful agents. It can therefore be assumed that the effect of these molecules derives from an intrinsic cytotoxicity towards non-self. Indeed, some studies have shown that molecules extracted from starfish, like Granulatoside D and Asterosaponins 1 and 3, exert a cytotoxic effect even on non-tumor cell lines, respectively, murine splenocytes [65], and rabbit erythrocytes [74].

## 22. Conclusions

As mentioned above, the growing interest to develop and employ new natural compounds in medical field arises from the need to obtain effective drugs against cancer, especially for those types that are currently intractable, and from the requirement for minimal adverse-side effects on human health. For this purpose, the ocean, thanks to its huge biodiversity and to the peculiar characteristics of secondary metabolites produced from marine organisms, constitutes an invaluable and promising resource for years now. Echinoderms constitute a big important source of bioactive molecules, as confirmed by the great number of studies performed by researchers during this decade. Sea cucumber are the most studied group and represent an excellent source of triterpene glycosides, the whose effects are widely confirmed in the literature [95,96,97]. Further, sea urchins have been the object of several studies, which have revealed the potential of biomolecules such as antimicrobial peptides [98,99,100]. Within this context, starfishes found clearly their considerable position among echinoderms because of the biological potential of their derived compounds. Moreover, a recent study reports for the first time the isolation and the structural characterization of a novel cysteine-rich antimicrobial peptide from an extract of the coelomic epithelium of the starfish *Asterina pectinifera* [101]. Sometimes, the comparison of marine-derived compounds with drugs typically employed to treat tumors has been surprising, as demonstrated, for example, by the study conducted by Mutee et al. [20] according to which the raw extract of *A. planci* was more effective compared to the chemotherapeutic drug Tamoxifen, commonly used against breast cancer. Moreover, some of the species from which the molecules have been extracted are edible, as in the case of sea urchins, sea cucumbers and even starfish that are included in the diet in some Asian countries, underlining the safety of natural substances compared to chemically synthesized. The discovery and development of marine-derived anticancer drugs enabled not only direct clinical benefits but also promoted the discovery of new mechanisms of action and molecular targets as associated scientific outcomes, making possible the development of alternative chemotherapeutic agents, the better understanding of the carcinogenic events and the subsequent progression of the disease [102]. To date, the studies carried out appear to provide solid bases to establish that starfishes, along with the other classes of echinoderms, could effectively contribute to the prevention and the treatment of several types of human cancer. However, further investigations are needed to get more insight into the mechanisms of action of the isolated biological compounds since most published works do not include any information about them. Furthermore, usually these compounds are tested only in vitro, and their biological properties could not be confirmed when tested in vivo. As regards sea stars, the only example of an in vivo study is that conducted by Du et al. [35], about the cerebrosides derived from the starfish *Asterias amurensis*. To our knowledge, there are no other cases in the literature to date. However, for other species of marine organisms, in vivo analyses have confirmed successfully the in vitro results, and thereby they would seem to present excellent opportunity. This is illustrated by the study of Steiner et al. [103] in which in vivo experiments in myeloma xenografts on chicken embryos revealed significant suppression of myeloma-induced neoangiogenesis close to xenografts and a reduced revascularization of xenografts following exposure to marine compounds, even at low concentrations. Other interesting examples are the study of Krishnan et al., [104] whose investigation evaluated the anticancer activity of crude methanolic extract of *Tetilla dactyloidea* (CMETD) on diethylnitrosamine (DEN) induced hepatocellular carcinoma in a rat model showing a potent anti-carcinogenic effect, and the recent study of Yuan and collaborators which evaluated the antitumor potentiality of TBL-12, a new sea cucumber extract, in xenograft prostate cancer mice in vivo [105]. Anyway, once established the pharmaceutical potential of marine compounds the unavoidable following step is their chemical synthesis. For instance, to date, several saponins derived from marine organisms have been synthesized, as recently reviewed by Xiao [106]. A critical issue in the developmental process of drug from marine organisms is the availability of adequate amount of the animals and their derived compounds without harming the marine environment. Indeed, the extraction of these biological products involves the sacrifice of the animals and the large-scale production of them would lead to the extinction of species and to a significant alteration of ocean ecosystems. Accordingly, the characterization of the molecular structures of the extracted compounds is certainly a priority, in order to obtain the molecules through organic synthesis in the laboratory, with the aim to give an important contribution to the medical field while ensuring the safeguard of biodiversity. Moreover, the progress of genetic engineering, whose purpose is to transfer the genetic information of the desired compound in the host cells, provides a better control of the isolation and the expression of genes of the marine organisms, helping to have a more targeted approach in the development of lead compounds from the marine environment [107].

## Figures and Tables

**Table 1:** Taxonomy of starfishes and the extracted molecules evaluated for their biological activities

Species	Bioactive Molecules
**Order Paxillosida** **Family Astropectinide**	
*Astropecten polyacanthus*	Steroids: Astropectenols A–D 5α-cholest-7-ene-3β,6α-diol 5α-cholest-8(14)-ene-3β,7α-diol 5α-cholest-7,9(11)-diene-3β-ol Glycosylated polyhydroxysteroid: Polyacanthoside A Triseramide (20R,24S)-3β,6α,8,15β,24-pentahydroxy-5α-cholestane Marthasteroside B Psilasteroside
*Astropecten monacanthus*	Asterosaponins: Astrosteriosides A–D; Psilasteroside; Marthasteroside B
*Craspidaster hesperus*	Polyhydroxysteroidal glycosides: Hesperuside A–C Novaeguinoside A
**Family Ctenodiscidae**	
*Ctenodiscus crispatus*	(22E,24ξ)-26,27-bisnor-24-methyl-5α-cholest-22-en 3β,5,6β,15α,25-pentol 25-O-sulfate (22E,24R,25R)-24-methyl-5α-cholest-22-en-3β,5,6β,15α,25,26-hexol 26-O-sulfate (28R)-24-ethyl-5α-cholesta-3β,5,6β,8,15α,28,29-heptaol-24-sulfate (25S)-5α-cholestane-3β,5,6β,15α,16β,26-hexaol Δ7-sitosterol
**Order Forcipulatida** **Family Asteriidae**	
*Asterias amurensis*	Cerebrosides
*Leptasterias ochotensis*	Asterosaponins: Leptasteriosides A–F Asterogenins: (23S)-6α,23-Dihydroxy-5 α-cholesta-9(11),20(21)-dien-3 β-yl sulfate, sodium salt; (22E)-6α-Hydroxy-5 α-cholesta-9(11),20(22)-dien-23-one-3 β-yl sulfate, sodium salt Sulfated steroid monoglycosides: Leptaochotensosides A–C Sulfated polyhydroxylated steroid: (24S)-5α-cholestane 3β,6β,15α,24-tetraol 24-O-sulfate
*Lethasterias fusca*	Lethasteriosides A, B Thornasteroside A Anasteroside A Luidiaquinoside
**Order Spinulosida** **Family Echinasteridae**	
*Echinaster luzonicus*	Luzonicoside A, Luzonicoside D
*Henricia leviuscula*	Leviusculoside G
**Order Valvatida** **Family Acanthasteridae**	
*Acanthaster planci*	Plancitoxin protein Asterosaponin Acanthaglycoside G Pentareguloside G Acanthaglycoside A Maculatoside (or luidiaglycoside B) Plancisides A–C
**Family Archasteridae**	
*Archaster typicus*	Archasterosides A, B Regularoside A
**Family Asterinidae**	
*Asterina pectinifera*	(25S)-5α-cholestane-3β,6α,7α,8,15α,16β-hexahydroxyl-26-O-14′Z-eicosenoate (25S)-5α-cholestane-3β,6α,7α,8,15α,16β,26-heptol (25S)-5α-cholestane-3β,4β,6α,7α,8,15α,16β,26-octol (25S)-5α-cholestane-3β,4β,6α,7α,8,15β,16β,26-octol Cholest-7-en-3-sodium sulfate (24S)-5α-cholestane-3β,6α,8,15α,24-pentol Asterosaponins P1-2 Cyclin B Polysaccarides
**Family Asteropseidae**	
*Asteropsis carinifera*	Steroidal biglycosides: Cariniferosides A–F Asterosaponins: Asteropsiside A, Regularoside A, Thornasteroside A
**Family Goniasteridae**	
*Hippasteria phrygiana* (*H. kurilensis*)	Hippasteriosides A–D Phrygiasterol Borealoside C
**Family Ophidiasteridae**	
*Certonardoa semiregularis*	Certonardosterols Certonardosides A–J Certonardosides K–N Certonardosides P2 and I3
*Narcissia canariensis*	Glycosphingolipids: Ophidiacerebrosides
**Family Oreasteridae**	
*Anthenea chinensis*	Polyhydroxysteroidal glycosides: Anthenoside A, Anthenosides B–K
*Anthenea sibogae*	Anthenosides S1–S6
*Anthenea aspera*	Anthenosides E, G, J, K, S1, S4, S6 Anthenosides A1 and A2
*Choriaster granulatus*	Granulatosides D and E, Linckoside L4, Echinasteroside B, Echinasterosides C, E and F, desulfated Echinasteroside A, 22,23-Dihydroechinasteroside A, desulfated Echinasteroside B, Linckoside B, Linckoside E, Linckoside F, Laeviuscoloside D, Granulatoside A, Steroid Heptaol
*Culcita novaeguineae*	Asterosaponins: Novaeguinosides I and II, Regularoside B Asterosaponin 1, Asterosaponin 2, Asterosaponin 3, Novaeguinosides A–D, Novaeguinoside E Polyhydroxy steroidal glycosides: Culcinosides A–D, Linckoside B, Halityloside A, Halityloside B, Culcitoside C5, Halityloside D, Halityloside E, Echinasteroside C, Linckoside F, Linckoside L
*Pentaceraster gracilis*	Pentacerosides A and B Nodososide (5α,25S)-cholestane-3β,6α,815β,16β,26-hexol 3-O-[(2-O-methyl)-β-D-xylopyranoside] Maculatoside Protoreasteroside

**Table 2:** Steroidal glycosides that exhibit the highest anticancer activity against various tumor cell lines.

Compounds	Species	Tumor Cell Lines	Mechanism of Action	IC50
Crude extract	*Acanthaster planci*	Human breast cancer MCF-7 cell lines	Induction of apoptosis	15.6 µg·mL^−1^
Plancitoxin I		A375.S2 melanoma cells	Inhibition of cell growth, induction of apoptosis mediated by mitochondrial membrane and p38 pathway	5.67 µg·mL^−1^
Butanol fraction		A375.S2	Induction of apoptosis and necrosis	112.65 µg·mL^−1^
Anthenoside A	*Anthenea chinensis*	human leukemia K-562, hepatoma BEL-7402, glioblastoma U87MG cells	-	
Anthenosides B–K		K-562, BEL-7402 cells	-	
Anthenosides J–K	*Anthenea aspera*	human melanoma RPMI-7951, breast adenocarcinoma T-47D, colorectal carcinoma HT-29	Induction of apoptosis	89, 91, and 85 µM
Archasterosides A	*Archaster typicus*	human cancer HeLa cells, mouse epidermal JB6 P+ Cl41 cells	-	24 μM
Archasterosides B		human cancer HeLa cells, mouse epidermal JB6 P+ Cl41 cells	Induction of p53- and AP-1-dependent transcriptional activities	14 μM
Regularoside A		human cancer HeLa cells, mouse epidermal JB6 P+ Cl41 cells	-	110 μM
Methanol extract 5α-cholest-7,9(11)-diene-3β-ol	*Astropecten polyacanthus*	HL-60 leukemia cells, PC-3 prostate cancer cells	Induction of apoptosis via regulation of apoptosis-related proteins and via the down-regulation of ERK1/2 pathway and C-myc	8.29 µg·mL^−1^ 25.42 µg·mL^−1^ 2.70 μM
(20R,24S)-3β,6α,8,15β,24-pentahydroxy-5α-cholestane		HepG2 (hepatoma cancer), KB (epidermoid carcinoma), LNCaP (prostate cancer), MCF7 (breast cancer), SK-Mel2 (melanoma)	-	18.03–21.59 μM
Methanol extract Astrosterioside D	*Astropecten monacanthus*	HL-60, PC-3, SNU-C5 colorectal cancer cells	Induction of apoptosis via the inactivation of PI3K/AKT and ERK 1/2 MAPK pathways and down-regulation of C-myc	0.84–3.96 µg·mL^−1^ 4.31–5.21 µg·mL^−1^
Cerebrosides (AAC)	*Asterias amurensis*	murine sarcoma cells (S180)	Induction of mitochondria-mediated apoptosis	216.36 μM
Peptides derived from the motifs of cyclin B	*Asterina pectinifera*	HCT-116 human colon adenocarcinoma and EC-9706 esophageal carcinoma cells	Induction of apoptosis	100 μM
(25S)-5α-cholestane-3β,4β,6α,7α,8,15β,16β,26-octol Asterosaponin P1 Cholest-7-en-3-sodium sulfate		HepG2, DLD-1, HCT 116, and HT-29 cells DLD-1, HCT 116, and HT-29 cells HepG2	- - Radiosensitizing activity through apoptosis induction	0.2 μM 150 μM 4 μM 1.6 μM
Fermented *A. pectinifera* with mushroom mycelia *C. militaris* (FACM)		B16F10 murine melanoma cells	Induction of apoptosis	0.25–0.2 µg·mL^−1^
Regularoside A Thornasteroside A	*Asteropsis carinifera*	T-47D human breast cancer, RPMI-7951 human malignant melanoma, HCT-116 human colon cancer cells	-	169, 117, 142 μM 82, 35, 70 μM
Certonardosterols	*Certonardoa semiregularis*	A549, SK-OV-3, SK-MEL-2, XF498, HCT15	-	-
15-keto sterol		Unspecified	-	-
Certonardoside C		SK-MEL-2		3.8 µg·mL^−1^
Certonardosides L		A549 SK-OV-3 SK-MEL-2 XF498 HCT15	-	7.5, 6.8, 5.8, 6.4, 3.9 µg·mL^−1^
Certonardosides M		A549 SK-OV-3 SK-MEL-2 XF498 HCT15	-	>30, >30, 9.7, 25.4, 43.4 µg·mL^−1^
Certonardosides N		A549 SK-OV-3 SK-MEL-2 XF498 HCT15	-	8.0, 8.4, 7.7, 7.2, 8.2 µg·mL^−1^
Certonardosides P2		SK-MEL-2	-	-
Granulatosides D, Echinasterosides F, desulfated Echinasteroside B Laeviuscoloside D	*Choriaster granulatus*	Murine splenocytes	-	4.7 ± 1.2 4.6 ± 0.3 4.5 ± 0.4 2.2 ± 0.3μM
Hesperuside A	*Craspidaster hesperus*	leukemia MOLT-4, hepatoma BEL-7402 human lung cancer A-549	-	3.62 ± 1.08 2.59 ± 0.94 5.26 ± 0.36µM
Hesperuside B		leukemia MOLT-4, hepatoma BEL-7402 human lung cancer A-549	-	1.84 ± 0.65 0.68 ± 0.12 2.67 ± 0.54
Hesperuside C		leukemia MOLT-4, hepatoma BEL-7402 human lung cancer A-549	-	2.40 ± 0.73 2.12 ± 0.81 5.72 ± 0.82 μM
(25S)-5α-cholestane-3β,5,6β,15α,16β,26-hexaol	*Ctenodiscus crispatus*	HepG2, U87MG	Inhibition of cell growth and induction of apoptosis	10–200 μM
Asterosaponin 1	*Culcita novaeguineae*	U87MG cells	Inhibition of cell growth	4.3 µg·mL^−1^
Asterosaponin 1 Asterosaponin 3		K-562, BEL-7402	-	3.57 µg·mL^−1^; 2.55 µg·mL^−1^; 3.75 µg·mL^−1^; 1,89 µg·mL^−1^;
Novaeguinosides A		K-562, BEL-7402	-	3.0 ± 0.6, 2.4 ± 0.3 µM
Novaeguinosides B		K-562, BEL-7402	-	7.9 ± 1.5, 9.5 ± 1.1 µM
Novaeguinosides C		K-562, BEL-7402	-	1.3 ± 0.2 0.7 ± 0.1µM
Novaeguinosides D		K-562, BEL-7402	-	4.6 ± 0.5 4.1 ± 1.0µM
Culcinoside A		human glioblastoma cell lines (U87, U251, and SHG44)	-	9.35 ± 0.46 11.28 ± 0.65 8.04 ± 0.32µM
Phrygiasterol	*Hippasteria phrygiana*	Ehrlich carcinoma cells	Inhibition of growth	50 µg·mL^−1^
Leptasterioside A Leptasterioside B Leptasterioside C	*Leptasterias ochotensis*	T-47D cells	-	2 10 23 μM
Ophidiacerebrosides	*Narcissia canariensis*	KMS-11 multiple myeloma, HCT-116 colorectal adenocarcinoma GBM glioblastoma multiforme	-	~20 μM
Maculatoside	*Pentaceraster gracilis*	Hep-G2 SK-Mel2	-	16.75 ± 0.69 μM 19.44 ± 1.45 μM

**Table 3:** Other effects of biological active compounds from starfishes against human cancer cells.

Compound	Biological Effect
CAV (protein toxin from the venom of *A. planci*)	Anti-proliferative effect on A375.S2 cell line
Acanthaglycoside A Maculatoside	Inhibition of colony formation of HT-29 and MDA-MB-231 cell lines; prevention of the migration of MDA-MB-231 cells
Anthenosides A2	Reduction of T-47D cell colony number
Polysaccharides (from *A. pectinifera*)	Chemopreventive effect on human colon adenocarcinoma and human breast cancer cells
Cariniferoside F, Halityloside A 6-O-sulfate, 4′-O-methylhalityloside A 6-O-sulfate	Inhibition of colony formation of RPMI-7951 and T-47D cell lines
Regularoside A Thornasteroside A	Inhibition of colony formation of HCT-116, RPMI-7951 and T-47D cells
Luzonicoside A	Inhibition of proliferation, migration and colony formation of RPMI-7951 and SK-MEL-28 cell lines
Hippasterioside D	Reduction of colony number and colony size of HT-29 cells
Leptasterioside A	Inhibition of colony formation of T-47D cells
Leptaochotensoside A	Reduction of colony formation of T-47D cells
Lethasteriosides A	Inhibition of colony formation of T-47D, HCT-116 and RPMI-7951 cell lines
